# The *Total Worker Health*® (TWH) approach: a systematic review of its application in different occupational settings

**DOI:** 10.1186/s12889-024-19500-y

**Published:** 2024-07-30

**Authors:** Veruscka Leso, Michele Carugno, Paolo Carrer, Fabio Fusco, Marco Mendola, Mariagaia Coppola, Salvatore Zaffina, Reparata Rosa Di Prinzio, Ivo Iavicoli

**Affiliations:** 1 Department of Public Health, Section of Occupational Medicine, University of Naples Federico II, Via S. Pansini 5, 80131 Naples, Italy; 2 Department of Clinical Sciences and Community Health, University of Milan, Via San Barnaba 8, 20122 Milan, Italy; 3 Epidemiology Unit, Fondazione IRCCS Ca’ Granda Ospedale Maggiore Policlinico, Via San Barnaba 8, 20122 Milan, Italy; 4 Occupational Health Unit, Fatebenefratelli-Sacco University Hospital, Via G.B. Grassi 74, 20157 Milan, Italy; 5 Department of Biomedical and Clinical Sciences, University of Milan, Via G.B. Grassi 74, 20157 Milan, Italy; 6 Occupational Medicine Unit, IRCCS Bambino Gesù Children’s Hospital, Piazza S.Onofrio, 4, Rome, 00165 Italy; 7 Alta Scuola Di Economia E Management Dei Sistemi Sanitari (ALTEMS), Università Cattolica del Sacro Cuore, Largo Francesco Vito 1, Rome, 00168 Italy

**Keywords:** Workers well-being, Health promotion, Hierarchy of controls, Occupational risk management, Occupational health, Occupational safety and health, Leadership commitment, Participatory approach, Integrated programs, Workplace policies and programs

## Abstract

**Background:**

The National Institute for Occupational Safety and Health (NIOSH), in 2011, developed the “*Total Worker Health*®” (TWH) as a holistic approach to protect and promote the workers’ safety, health, and well-being. After over ten years from the TWH development, the aim of the present systematic review is to provide a comprehensive overview of the worldwide TWH initiatives.

**Methods:**

PubMed, Scopus and ISI Web of Science were searched for TWH studies published up to the 31^st^ of July 2023, and 43 investigations could be included. The review was registered on the International prospective register of systematic reviews PROSPERO with the reference number CRD42023416972.

**Results:**

Issues that emerged as relevant for the TWH operationalization were the awareness about the TWH approach and fundamentals, the leadership commitment, and a participatory engagement of the workforce: these aspects all contributed to acceptable and effective setting oriented TWH plans, specifically tailored on the peculiarities of the workplace, including small enterprises and multiemployer worksites. Evaluation and continual improvement were reported as fundamental for the successful implementation of TWH initiatives. Limited resources for safety and health initiatives, in terms of time, people, and funds, together with difficulties in the identification of safety and health priorities and a poor participatory culture were recognized as obstacles to the TWH application. Training resulted the core component of the TWH leadership and workforce preparedness, with beneficial results in terms of safety culture and adoption of preventive measures.

**Conclusions:**

Although interesting aspects emerged from our review, future longitudinal investigations should confirm the effectiveness, easy integration, and long-term sustainability of TWH models in different workplaces, in order to effectively support safe and health-enhancing works able to improve innovation and productivity.

## Introduction

The National Institute for Occupational Safety and Health (NIOSH), in 2011, defined “*Total Worker Health*®” (TWH), as “policies, programs, and practices that integrate protection from work-related safety and health hazards with promotion of injury and illness-prevention efforts to advance worker well-being.” A hazard-free work environment is among the priorities of the TWH approach that, through the integration of workers’ safety, health, and well-being, aims to improve their creativity, innovation, and productivity [1].

In fact, in todays’ workplaces, the emergence of innovative patterns of employment, the widespread use of precarious and part-time work arrangements, the global competition for workers, products, services, and knowledge, as well as the increasing demographic workforce diversity, including gender differences and aging, may all impact workers’ health and well-being, requiring successful health and productivity management that may benefit from an implementation of the TWH approach [2].

However, this aim requires facing some challenging issues, such as the need for leadership commitment and suitable strategies for employee engagement; organizational policies and practices; supportive benefits and incentives; accountability and training; as well as integrated real-time evaluation and monitoring plans providing guidance for improvement actions [1].

Therefore, to assist organizations in launching and maintaining TWH programs, the NIOSH Office for TWH, in 2016, published the Hierarchy of Controls Applied to NIOSH TWH, adapting the framework used in traditional Occupational Safety and Health (OSH) to the operationalization of the TWH model [3]. Such strategy includes five levels of controls in descending order of likely effectiveness and protectiveness: 1. removing working conditions that lead to or exacerbate worker illness and injury or that adversely affect well-being; 2. substituting policies, programs, and management techniques that strengthen the workplace culture of safety and health; 3. redesigning the workplace as necessary for safety, health, and well-being; 4. giving all employees resources and instructions on safety and health; 5. providing assistance to employees with personal risks and challenges, promoting healthier decision-making, and encouraging personal change [4, 5].

Recognizing that the experiences of work and outside work can act together to produce worker illness and injury, the TWH approach may help businesses and communities in reducing the impact and cost of such adverse events (thereby controlling healthcare costs and the impairment to family and community life) and advancing workers’ well-being in ways that support a full, rewarding life [6, 7].

To increase awareness among the TWH principles and their application, several toolkits, actionable guidance, web-based training, continuing education courses, and other practice-based resources have been developed. However, although the TWH approach can have some global reach, its worldwide operationalization requires to consider differences related to the country’s incomes and cultures, businesses type and dimension as well as to the various national OSH preventive policies and programs adopted [8].

In this context, after over ten years from the TWH development, it seems crucial to conduct a thorough evaluation of the TWH operationalization in different occupational settings to overcome knowledge gaps and derive valuable information able to guide the future dissemination and employment of standardized TWH models. Therefore, the present review aims to revise available literature information on TWH initiatives, strategies, fields of intervention, outcomes, and efficacy evaluation in various worldwide workplace realities. Our final aim is to achieve a comprehensive and critical overview on TWH-related issues that may support the design and implementation of future initiatives finalized to protect workers' health and safety and promote their well-being through the creation of work and work environments that are safe, health-enhancing, meaningful and fulfilling.

## Materials and methods

A systematic review was performed according to the Preferred Reporting Items for Systematic Reviews and Meta-Analyses Statement (PRISMA) criteria [9]. The review was registered on the International prospective register of systematic reviews PROSPERO on the 2^nd^ of May 2023 with the reference number CRD42023416972.The PubMed, Scopus and ISI Web of Science databases were searched to identify studies, published up to the 31^st^ of July 2023, focusing on the application of the TWH approach in different occupational scenarios, and addressing strategies, fields of intervention, outcomes, and effectiveness of TWH initiatives. The search term “Total Worker Health” was employed in each database. The choice to use such a general search term was motivated by the need to avoid loss in suitable publications. All the titles and abstracts retrieved were independently analyzed by three of the authors who selected relevant papers. Inclusion criteria regarded cross-sectional, longitudinal, cohort, and case control studies, published in English and exploring initiatives of health protection/promotion developed according to the NIOSH TWH approach in different occupational settings both referring to the “in field” analysis of the fundamentals for the operationalization of the TWH model, as well as on the concrete application of standardized TWH programs in different workplace realities. Review articles, book chapters, conference papers, letters to editors, editorials, commentaries, and out of topic papers were excluded. Exclusion criteria regarded also studies describing workplace health promotion interventions, not referring to TWH, as well as those papers focusing only on the theoretical fundamentals of TWH or describing exclusively the rationale and methods for a possible TWH implementation, without reporting a concrete application of the model on specific populations. In the “Results'' section, and in Tables 1 and 2, the findings of the studies have been summarized, including information on the characteristics of the working populations explored such as sex, mean age, occupational sector, and tasks performed. Specific TWH programs adopted were reported, with a focus on the strategies applied in mapping the needs and putting in action the interventions at various organizational levels, explicitly referring to the different steps of the NIOSH hierarchy of controls model described for the TWH operationalization. When available, the effectiveness of the initiatives has been also summarized in terms of health outcomes, as well as company and workforce benefits.

**Table 1:** Exploratory studies

Study Location	Occupational sector and workers’ characteristics	Intervention features	Outcomes	Reference
USA	Real estate workers (*n* = 60); Age (mean): 41; Gender: not applicable (NA)	*Healthy Workplace Participatory Program (HWPP)* Mapping of needs; Design of intervention Focus group	Identified participatory program as an opportunity to suggest improvements to their workplace through workers’ own efforts; Identified topics for priority intervention: improving communication between staff and management, ability to collaborate effectively on workplace change efforts, management awareness of problems	Robertson et al. [10]
New England, USA	Miscellaneous of workers (*n* = 950); Age: NA; Gender: NA	*Healthy Workplace Participatory Program (HWPP)* Mapping of needs, barriers, and facilitators; Design of intervention Focus group + surveys	Identified health/safety issues, physical and psychological concerns, work-related stress as priority issue to address, and maintaining a good health and safety climate as a factor promoting success, using focus groups and surveys as instrument to identify priority areas of intervention; At 2 years follow-up, reported high engagement and satisfaction using a participatory program, gaining of new skills in problem solving, decision making and team working, and improvement in managers' understanding of health and safety issues;	Nobrega et al. [11]
Boston, USA	Managers and workers in the healthcare sector (4 units); Age: NA; Gender: NA	Application of an inspection tool to identify hazards and ways to mediate ergonomic risk factors	Importance to measure work practices to improve organizational programs and policies, for example inherent to collision hazard areas and hazards involving posture, lifting, pushing, and pulling, and to track progress over time	Grant et al. [12]
USA	Managers and workers in the healthcare sector (*n* = 58); Age: NA; Gender: NA	Mapping of needs Focus group	Importance to improve leadership support, employees' safety, open communication; Suggestions about access to employee gym, smoking cessation and management programs, more walking events, creation of support groups for employees' chronic disease management	Schult et al. [13]
USA	Miscellaneous of managers (*n* = 14); Age: NA; Gender: NA	*Workplace Integrated Safety and Health (WISH)* Mapping of facilitators Focus group	Identified topics to protect and promote workers safety, health, and wellbeing: leadership commitment, participation, policies, programs, and practices for supportive working conditions, collaborative strategies, adherence to ethical norms, data-driven change	Sorensen et al. [14]
USA	Home care workers (*n* = 28); Age (mean): 48.8; Gender: M 16.7%, F 83.3%	*Community of Practice and Safety Support (COMPASS)* Qualitative follow-up Meetings	Declared improvement on safety awareness, use of injury-reducing postures and tools, communication with consumer-employer regarding workplace hazards. Positive feedback in professional contacts and collaborative problem solving with peers	Mabry et al. [15]
Kentucky, USA	Safety managers of aluminum rolling plant sector (*n* = 2); Age: NA; Gender: NA	*Harvard Integration Instrument* Mapping of barriers; Design of intervention Surveys + interviews + toolkit + training	Barriers: job/production demands, attitudes and knowledge level of new employees and employees’ mindset outside workplace, lack of time devoted to meet workers together; Declared increase of safety manager's awareness of TWH concepts and understanding of needs to integrate programs, policies, and practices	Watkins et al. [16]
Washington (USA)	Line, mid-level, executive-level managers of state agencies employing workers representing a variety of job titles (*n* = 23); Age: NA; Gender: M 57%, F 43%	Mapping of barriers and facilitators Interviews + surveys	Barriers: wellness culture is not integral to all agencies, perceived lack of employee buy-in, lack of supportive messages from manager above, lack of awareness of agency policies, scheduling inflexibility, workload Facilitators*:* Awareness of wellness activities and resources; Regular communications from the wellness coordinators or related staff; Wellness supportive culture: allocation of money, staff time, active promotion through emails, employee intranet, posters/signage, announcements in staff meetings and all staff broadcasts; Perceived benefits of wellness programs (lower stress and burnout; increased productivity; lower healthcare costs; lower absenteeism): Employees’ view of the wellness program; Role expectations and modeling; Comfortable talking about wellness; Awareness of the agency’s policies regarding workers’ participation in wellness programs; Scheduling flexibility balancing workload to accommodate participation,	Passey et al. [17]
Iowa and Nebraska, USA	Miscellaneous of workers of 32 small businesses; Age: NA; Gender: NA	Mapping of needs and facilitators Interviews	Facilitators individuated to enhance health and safety practices, policies and programs in small enterprises: top management support, multilevel leadership engagement, a participatory approach and policy development for a long-term organizational change, tailoring programs to meet the needs and preferences of workers; Needs identified by employers: to analyze existing programs, policies and practices as well as to examine relevant data in order to prioritize needs and direct resources accordingly, and to implement low-cost strategies defining suitable qualitative and quantitative evaluation metrics	Rohlman et al. [18]
Colorado and Wyoming, USA	Miscellaneous of managers of 18 small businesses; Age: NA; Gender: NA	Mapping of barriers Interviews	Barriers identified: offsite workforce, lack of employee’s engagement, generational differences (e.g., millennials being perceived as unreliable and not working as hard as older generations)	Thompson et al. [19]
USA	Retail setting workers (*n* = 120); Age (mean): 42; Gender: M 47.6%, F 52.4%	*Healthy Workplace Participatory Program (HWPP)* Mapping of needs; Design of intervention Focus group + surveys + online tool	Identified topic areas of need and interventions’ component as diet at work and health awareness; Participatory process as opportunity for more open dialogue with management	Strickland et al. [20]
Colorado, Oregon, Florida, USA	Miscellaneous of managers and workers of 382 small and large businesses; Age: NA; Gender: NA	*Health Links (Healthy Workplace Assessment)* Mapping of facilitators Surveys	Larger businesses demonstrated a more comprehensive approach to health and safety based on: organizational supports, health insurance, companywide communication, workplace assessments on health risks, employee needs and interests, health and safety education, incentives to increase employee participation, implementation of programs about changes in the way to work to reduce injuries, providing of personal protective equipment and plans for disaster and emergency preparedness, engineering and administrative controls, health policies and program about physical activity, stress management, nutrition, tobacco control, mental health	Tenney et al. [21]
Massachusetts, USA	Managers and workers of construction sector (*n* = 93); Age: NA; Gender: NA	*Harvard Center (conceptual model) for Work health and Well-being Guidelines* Mapping of needs: Design of intervention Focus group + interviews	Identified topic areas of needs and intervention components as chemical exposure, lack of communication for safety concerns, lack of workplace smoking policies, stigma related to accommodating health/injury at work, health concerns related to meal break policies and lack of dedicated area; Designing leadership training identifying priorities related to communication, and workforce participation including a manual and accompanying materials to guide activities, providing resources, communication between frontline workers and managers, prioritizing problematic working conditions identified by workers, implementing changes to company policies, programs and practices, and participatory needs assessment	Peters et al. [22]
USA	Managers and workers of correctional sector (*n* = 401); Age (mean): 44; Gender: M 48%, F 52%	*Community-Based Participatory Research (CBPR)* Mapping of needs Focus group + surveys + interviews	Identified topics for priority intervention: workplace culture, communication with leadership, training, safety, community; Identified participatory program as an opportunity to voice their concerns	Jaegers et al. [23]
USA	Correctional workers (*n* = 221); Age: NA; Gender: NA	*National Corrections Collaborative (NCC)* Mapping of needs Meetings	Identified necessity to participate in intervention and reform activities, to create an alliance with research to identify needs and solutions, to highlight mental health questions, to develop a TWH collaborative approach involving researchers and specialists to create solutions	El Ghaziri et al. [24]
Colorado, USA	Miscellaneous of 53 organizations (*n* = 1271); Age (mean): 41.3%; Gender: M 33%, F 66.2%	*Health Links (Healthy Workplace Assessment)* Mapping of needs Interviews + surveys + in-person training and virtual follow-up	Identified leadership commitment as positively correlated with safety climate and with participatory safety behaviors	Shore et al. [25]
Colorado, USA	Miscellaneous of supervisors and workers of 36 small businesses (*n* = 1052); Age (mean): 40.30; Gender: M 35.83%, F 63.94%, Other 0.22%	*Employee Health and Safety Culture Survey* Mapping of facilitators Surveys	Identified that small businesses seeking to engage employees in TWH effort should build strong safety and health climates because of their influence on employees’ motivation to participate in health promoting and health protective programs	Schwatka et al. [26]
USA	Managers and workers in the healthcare sector (*n* = 78); Age: NA; Gender: M 50%; F 50%	*Healthy Workplace Participatory Program (HWPP)* Mapping of barriers and facilitators Surveys + interviews	Barriers: limited resources, difficulty to individuate safety and healthy priorities, poor participatory culture; Facilitators: to support leaders toward a TWH approach, to help workers to know better their proactive role and the importance of positive communication methods in a safety and health program via communication tools (announcements, updates, newsletters)	Nobrega et al. [27]
Massachusetts, USA	Managers in the food service sector (*n* = 62); Age: NA; Gender: NA	*The Workplace Organizational Health Study* Mapping of needs, barriers, and facilitators Interviews	Identified leadership support as necessary to prioritize worker safety and health improving working conditions as safety and ergonomics, work intensity, job enrichment; Barriers: financial pressures, competing priorities, fast-paced work environment, unsure availability of resources, lack of communication between site managers and district leaders; Facilitators: leadership support, reinforcing of the need for accountability, coaching and feedback	Sorensen et al. [28]
Guatemala, Nicaragua, Mexico, Latin America	Managers and workers in the agricultural sector (*n* = 517); Age: NA; Gender: NA	*Health Links (Healthy Workplace Assessment)* Mapping of needs Focus group + interviews + online tool	Identified topic areas of needs and intervention components: improvement of a safety and wellness culture, chronic disease prevention, stress and mental health management, sleep hygiene and fatigue management	Jaramillo et al. [29]
USA	Miscellaneous of managers of small businesses (*n* = 11); Age: NA; Gender: NA	*Small Business Intervention Diffusion Model* Mapping of needs Focus group + interviews + phone calls	Identified that wellness issues are harder to address than workplace safety, thus emphasize benefits to the company’s bottom line is needed; Identified need to design TWH interventions flexible and tailored to the individual business; Limited resources as a challenging issue for TWH operationalization; In health and safety programming critical topics were: recognition and personal relationships; Identified the importance of use of intermediaries that provide goods or services to assist small business in health and safety concerns	Cunningham et al. [30]
USA	Miscellaneous of workers (*n* = 13); Age: NA; Gender: NA	Mapping of facilitators and barriers Interviews	Identified key issues favoring the TWH operationalization: leadership commitment, available resources especially to support organizational tobacco control efforts and to address sedentary work, improvement of organizational efforts focused on the prevention of work-related stress, fatigue, and sleep	Hudson et al. [4]
Colorado, Oregon, USA	Miscellaneous of workers of 200 small and medium businesses; Age: N/A; Gender: N/A	*Health Links (Healthy Workplace Assessment)* Mapping of facilitators Surveys	Identified the TWH consultations as a key element to enhance the adoption of organizational behaviors that promote workers’ health, safety, and well-being over time	Tenney et al. [31]
USA	Managers of the mining industry (*n* = 13); Age: NA; Gender: M 84.6%, F 15.4%	Mapping of needs Focus group	Identified the importance of benefits of having a positive safety culture, and topics as enhanced communication with leadership, caring leadership, family atmosphere as elements of a safety culture	Smith et al. [32]
USA	Managers in the correctional sector (*n* = 157); Age (mean): 42; Gender: M 77.7%, F 22.3%	*Healthy Workplace Participatory Program (HWPP)* Mapping of needs Survey	Identified topics for priority intervention: improving sleep quality/quantity, healthy eating and healthy emotional expression, reducing stress, alcohol/substance use, caffeine use, high blood pressure/cholesterol and musculoskeletal pain, and increasing physical activity, preventing chronic diseases	Dugan et al. [33]
Northeastern, USA	School workers (*n* = 14); Age: NA; Gender: M 7.1%, F 92.9%	*Healthy Workplace Participatory Program (HWPP)* + *All Employee Survey (AES)* Mapping of needs, barriers, and facilitators; Design of interventions Focus group + surveys	Identified topics for priority intervention: physical and psychological wellness, physical activity after a school day, involving of a human resource professional to manage health and safety programs and resources, training on stress management, than more involvement of workers, design of break moments, more positive feedback to workers, minutes to teachers after meetings as other declared needs; Barriers: stress related to low communication, lack of self-care, high workload, inadequate resources	Sanetti et al. [34]
Colorado, USA	Managers and workers of the construction sector (*n* = 150); Age (mean): 42 (managers), 36 (workforce); Gender: M 85.3%, F 14%, Other 0.7%	Mapping of needs Surveys	Identified promising effects of a “shared TWH transformational leadership”: safety and health climate, positive perceptions on safety and health by support from co-workers, more inspiration for employees to achieve their goals; A shared purpose for TWH amongst their teams and voice around TWH issues were positively associated with sharing of TWH transformational leadership responsibilities	Schwatka et al. [35]

**Table 2:** Applicative studies

Study Location	Study Design	Occupational sector and workers’ characteristics	Intervention features	Outcomes	Follow-up period	Reference
USA	Randomized control trial	Supervisors and workers of construction sector (*n* = 167); Age (mean): 45.13; Gender: M 90%, F 10%	*Safety and Health Improvement Program (SHIP)* Supervisor-based training on family supportive supervisory behaviors, safety climate, communication skill (team effectiveness)	Improvement in supervisors’ emotional support behaviors, team effectiveness increasing morals and working attitudes (90%), more efficient use of time and resources (70%), focus on safety practices (100%); Decrease of mean blood pressure scores (p = .038)	12-month	Hammer et al. [36]
USA	Pre-post-test	Home care workers (*n* = 16); Age (mean): 57.81; Gender: M 6%, F 94%	*Community of Practice and Safety Support (COMPASS)* Training on health and safety promotion, risks, social support for “consumer/employers”; Goal-based teamwork	Increase of life satisfaction (p < 0.05), decision authority, fruit and vegetable consumption, safety compliance, team cohesion; bringing meals from home to work; Decrease of negative affect (p < 0.05), depressive symptoms, job psychological demand, interpersonal conflict with customer-employers and occupational fatigue	6-month	Olson et al. [37]
USA	Randomized control trial	Overweight/obese workers working in sedentary desk jobs (*n* = 54); Age (mean): 45; Gender: M 30%, F 70%	Redesigning of work environment introducing elliptical machine underneath job desk; Advice on ergonomic strategies; Periodic recommendations by weekly mails on ergonomic strategies and healthy behaviors	Improvements in occupational physical activity counts (p = 0 .03), occupational time spent in light-intensity physical activity (p = 0.04); In relation to average (1) pedal time/day (min), (2) pedal bouts/day and (3) pedal speed HP/HP changes in weight (p = 0.04), fat mass (p = 0.02), % body fat (p < 0.05), resting heart rate (p < 0.05), waist circumference (p = 0.02), concentration while at work (p = 0.01), days missed because of physical/mental health (p = 0.03);	16-weeks	Carr et al. [38]
USA	Randomized control trial	Home care workers (*n* = 149); Age (mean): 51,6; Gender: M 11%, F 89%	*Community of Practice and Safety Support (COMPASS)* Training meets on safety, health, well-being, goal setting, self-monitoring, social support; Peer-oriented discussion meets	Improved safety communication (12-month p < 0.001); correcting slip, trip, or fall hazards (12-month p = .027); Using of new tools or technique for moving objects (6-month p = .009), house cleaning (6-month p = .041; 12-month p = .006) and daily fruit and vegetable servings (12-month p = 0.38); Reduction in lost work days because of injury (6-month p = 0.01), improvements in high-density lipoprotein (6-month p = 0.045) and grip strength (12-month p = 0.011)	6-month and 12-month	Olson et al. [39]
Boston, Massachusetts, USA	Randomized control trial	Construction workers (*n* = 324); Age (mean): 40.55; Gender: M 96%, F 4%	*All the Right Moves (ARM) program (Soft Tissue Injury Prevention program)* Foreman training on worksite ergonomic practices; Coaching during Health Weeks on diet, physical activity, reduced smoking behaviors	Increase of physical activity (6-month p = 0.03), healthier diet (6-month p = 0.008) and eating (6-month p = 0.054) behaviors, ergonomic practices (1-month p = 0.002); Reduction of new pain or injury (1-month p = 0.012)	1 and 6-months	Peters et al. [40]
Oregon, USA	Pre-post-test	Supervisors and workers of contractor sector (*n* = 35); Age (mean): 38; Gender: supervisors: M 90%, F 9.1%; workers: M 69.1%, F 30.8%	Computer-based training on supporting of employees' health lifestyles; Discussion meetings on health topics and take-home healthy activities	Increase of health knowledge (p < 0.001), family supportive supervisors’ behaviors toward employees (p = 0.005), safety climate (p = 0.054), social support for a healthy diet by family and others, strengthening and toning muscles, support to a healthier environment, sleep hours, vitality, general health. Decrease of sugary drink and sugary snack consumption, systolic blood pressure	14-weeks	Anger et al. [41]
USA	Pre-post-test	Sale workers (*n* = 70); Age (mean): N/A; Gender: M 52.86%, F 47.14%	*Promoting U through Safety and Health (PUSH)* Online training on safety, health, communication	Increase of knowledge of safety and health score (p < 0.001), specific skills for the job (mentioned by 17%), awareness of hazards and how to apply information learned (mentioned by 20%)	3-months	Aryal et al. [42]
Colorado, USA	Pre-post-test	Miscellaneous of managers of 22 small facilities; Age (mean): N/A; Gender: N/A	*Health Links (Healthy Workplace Assessment)* TWH assessment, advising and training for leaders on TWH principles	Increase of organizational supports, workplace assessments, health programs and policies, safety programs and policies, engagement, evaluations, health climate, safety climate	1-year	Shore et al. [43]
USA	Randomized control trial	Supervisors and workers of military sector (*n* = 704); Age (mean): 36.2; Gender: M 25.1, F 74.7	Online training on family and sleep supportive supervisory behaviors	Increase of employee's perceptions of supervisor support for sleep (p < 0.01), job satisfaction; decrease of turnover intentions, stress before bed, personal functional impairment, and social functional impairment	4-months and 9-months	Hammer et al. [44]
USA	Pre-post-test	Supervisors and workers of agricultural sector (*n* = 182); Age (mean): N/A; Gender: M 35.7%, F 63.7%, Ot. 0.55%	Online training on supervisors’ attitudes about health promotion and workers’ well-being	Increase of knowledge of safety and health risks and promotion scores, supervisors talking about safety and health to young workers (p < 0.001)	Immediate post-test and 3-months	Rohlman et al. [45]
New England, USA	Randomized control trial	Supervisors and workers of construction sector (*n* = 263); Age (mean): 44; Gender: M 97%, F 3%	*HearWell* Training on hearing risks, strategies for noise hazards and protection device use	Increase of self-efficacy in hearing protection devices (p = 0.04), social norms around hearing protection (p = 0.001), hearing climate (p = 0.005)	6-months	Cavallari et al. [46]
USA	Randomized control trial	Correctional workers (*n* = 128); Age (mean): 30.38; Gender: M 75.8%, F 24.2%	*Peer Health mentoring Program (HMP)* Peer mentoring on TWH principles and better healthy behavioral strategies	Decrease of perception of both physical and psychological job demands (p < 0.001)	1-year and 5-years	Kotejoshyer et al. [47]
Colorado, USA	Randomized control trial	Miscellaneous of managers of small businesses (*n* = 38); Age (mean): 42; Gender: M 8.8%, F 91.2%	*Health Links (Healthy Workplace Assessment)* Training on TWH strategies, leadership practices and personal health; Virtual coaching and goal tracking	Improved self-reported TWH leadership practices about well-being (p = 0.19) but not in their personal health reporting increased levels of work stress after program	3-month	Schwatka et al. [48]
Colorado, USA	Pre-post-test	Miscellaneous of leaders (*n* = 261); Age (mean): 38.5; Gender: M 20.7%, F 78.9%	*Health Links (Healthy Workplace Assessment)* TWH training for leaders; Coaching sessions, goal setting and tracking platform	Health climate and safety climate remained stable, but well-being scores declined in COVID I (p < 0.0001) and in COVID II (p < 0.001) time points	6-weeks and 4-month (COVID I and COVID II time point)	Brown et al. [49]
USA	Randomized control trial	Miscellaneous of managers of small businesses (*n* = 250); Age (mean): 40.8; Gender: M 25% males, F 75%	*Health Links (Healthy Workplace Assessment)* Training and advising on TWH practices	Increase of TWH policies and programs (p = 0.682), health leadership (p = 0.880), safety climate (p = 0.456), health behavior (p = 0.495), well-being (p = 0.071)	1-year	Schwatka et al. [50]
USA	Randomized control trial	Bus operators (*n* = 14); Age (mean): 49.57; Gender: M 64.29%, F 35.71%	*Success & Health Impacts For Transit (SHIFT) operators during Onboarding* Onboarding training on prevention of weight gain and functional skills oriented to a new workplace; Website application to support online challenges	Decrease of weight, sugary drink, sugary snack, fast food consumption and general work-related stress; Increase of meals bright from home, self-reported physical activity, self-reported sleep duration and sleep quality, social connection, self-efficacy, job satisfaction	3–6-9–12-months	Olson et al. [51]

## Results

**Fig. 1:**
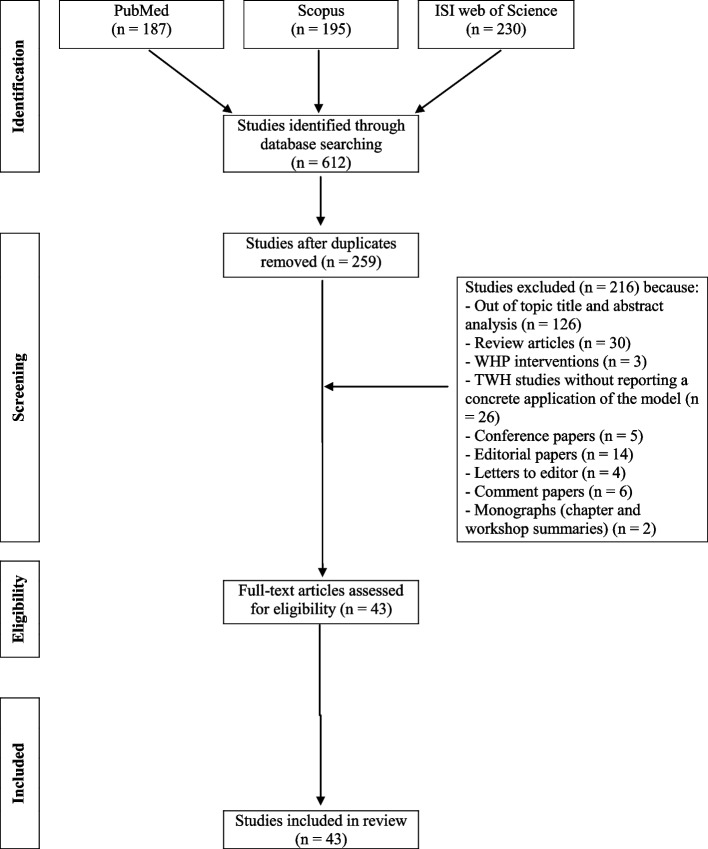
Flowchart of the literature search

The preliminary search performed on Pubmed, Sopus and ISI Web of Science databases retrieved 187, 195 and 230 references, respectively, for a total of 612 articles. Three-hundred-and-fifty-three duplicated studies were removed from the total of papers. Out of the remaining 259 articles, 216 were excluded because they did not meet the inclusion criteria based on title and abstract examination. In particular, 126 articles were excluded because out of topic, 57 because review articles or other types of papers not considered suitable for inclusion, 3 studies because reported health promotion intervention not referring to the TWH approach, 30 because focused on the theoretical fundamentals of the TWH model or addressed only the rationale and methods for a possible TWH approach application without reporting a concrete operationalization of the model. A careful analysis of the reference list accompanying all selected articles was performed, although no additional relevant publications could be added to the previously detailed literature search. Overall, a total of 43 studies were retrieved for the review purposes (Fig. 1). Regarding the methodology of the included studies, 27 were cross-sectional, while 16 were case–control investigations. As concerns their geographical location, most of the included studies (42) were performed in the USA, in line with the origin of the TWH model, and only one in Latin America. Retrieved studies included “exploratory” investigations (27), primarily focused at exploring favoring issues and obstacles to the operationalization of the TWH approach as well as to identify companies’ and workers’ needs with respect to safety, health, and well-being (Table 1) and “applicative” ones (16) (Table 2), that mainly described the application of TWH models in different occupational settings. The following paragraphs will analyze in greater detail the findings of the selected articles.

### Exploratory studies

The “exploratory studies” (Table 1), were generally conducted through focus groups, online surveys and interviews, with the aim to assess aspects relevant in promoting or preventing the operationalization of the TWH model in different occupational contexts. These may be important to identify elements that may guide setting-oriented programs to support and promote workers’ well-being.

#### General aspects promoting TWH initiatives

Total Worker Health programs represent a holistic approach for advancing worker safety, health, and well-being. Organizational readiness for such measures requires competencies in leadership, communication, subject expertise, and worker participation. The following paragraphs will detail elements that emerged as essential for a suitable and sustainable operationalization of the TWH approach in different occupational settings.

#### Awareness about the TWH approach and fundamentals

The recognition and awareness about the TWH approach and the hierarchy of controls, together with the inclusion of the TWH principles among the existing organizational values were reported as elements favoring the TWH operationalization [4]. To this aim, determining factors were the elimination/reduction of working conditions that could threaten safety, health, and well-being of workers; the adaptability and resources to substitute unhealthy working conditions; a redesign and education approach; an organizational culture aimed to encourage the promotion of healthier personal choices. Training, interviews, and surveys aimed at increasing the managers’ awareness on the TWH concepts and the comprehension of workers’ needs resulted essential to develop and implement suitable TWH programs, policies, and practices [16].

#### Leadership commitment

The leadership commitment was identified as a key element to protect and promote workers’ safety, health, and well-being [25, 27, 48]. To achieve this aim, managers of the mining industry underlined the importance to develop an open communication, a caring leadership, and a familiar atmosphere [32]. The leadership support and commitment to the intervention at its start resulted necessary to prioritize workers’ safety and health, ensure availability of resources, and reinforce the need for accountability also in the low-wage food service, characterized by physically demanding work, job insecurity, uncertainty around work hours, earnings instability, repetitive work, and low job decision latitude and autonomy [28, 52–55].

Leadership was essential also to support the employee participation in wellness programs [17, 27]. Comparably, participation of workers in well-being programs could be promoted by the assessment of employees’ view of the plans, the implementation of the leadership’s awareness regarding the well-being promotion and the potential managers’ role-modeling, together with comfortable talking, sharing information with employees about wellness, and scheduling flexibility aimed to balance workload to accommodate participation [17].

To favor the workforce engagement in TWH interventions, the leadership approach should move from a vertical, hierarchical conception to a “shared”, “transformational” one [35]. This approach has been demonstrated to be consistently related to better safety and health outcomes, as it was associated with the development of a stronger “multilevel TWH leadership identity” joined by employers and employees.

#### Workforce involvement

A participatory approach able to ensure that workers have been engaged in identifying company safety and health needs, contributing to the design of TWH programs, and participating in all aspects of program implementation and evaluation has been reported as relevant for a successful management of occupational risks and the promotion of the workforce health and well-being [10, 24]. In this view, some concerns have been expressed regarding the limited communication between frontline and managerial personnel [27], particularly evident in specific contexts, like the construction sector, where the industry’s structure and employment practices, characterized by multiple employers and subcontractors, make implementing integrated TWH approaches a challenging issue. A continuous improvement cycle through a communication infrastructure between frontline workers and managers could be considered as a possible overcoming solution [22].

However, an improvement of workforce skills useful for this purpose, such as suitable communication abilities [20, 22, 23] and skills in problem solving, decision making, and team working need to be promoted [11]. Furthermore, workers highlighted the need to improve their knowledge on safety and health, starting from the intrinsic features of their work and related risks [15].

#### Setting oriented TWH plans

The relevance of flexible and setting-oriented plans for the application of the TWH model emerged in various occupational realities [18, 30]. An enhanced collaboration between the leadership and the workforce was recognized as a transversal and central element to design suitable and tailored strategies e.g., in the mining [27], construction and real estate [10, 22], healthcare [13], and other sectors [14]. In specific contexts, like the construction sector, where multiple employers and subcontractors exist, low-cost solutions may characterize the win strategy for a suitable and timely implementation of TWH plans [22].

When the operationalization of the TWH model was explored in small enterprises, employers identified the need to analyze existing programs, policies, and practices as well as to examine relevant data to prioritize needs and allocate resources accordingly [18]. It was also apparent that the business size [21] and nature of the workplace dictated the type and format of programs, e.g., eliminating slip, trip, and fall in manufacturing facilities, or facing hazards associated with sedentary works in office ones [18]. Tailoring programs to meet the needs and preferences of workers was reported as a key element. All the employers indicated a strong need for effective low-cost strategies. Additionally, there was a clear interest in defining suitable qualitative and quantitative evaluation metrics. Additionally, small businesses should build strong safety and health climate because these may influence the employees’ motivation to participate in health protection and promoting programs [26]. Tenney et al. [31], demonstrated that the delivery of TWH advising was able to enhance the adoption of organizational behaviors promoting workers’ health, safety, and well-being over time in small-to-medium businesses. Cunningham et al. [30] demonstrated that the use of intermediaries, or organizations that provide goods or services to small businesses, e.g., insurers, health providers, government agencies, suppliers, trade associations, and chambers of commerce, could be an effective way of reaching small employers with health and safety assistance. In addition, programs and initiatives bringing different small businesses together to pool resources and share events could be successful. They confirmed the need for TWH programs flexible and tailored to the individual occupational context. Scaling out, in this perspective, may be useful to adapt and deliver evidenced based interventions to new populations or in a new delivery system to increase fidelity, acceptability, understanding, feasibility, system alignment, and leader engagement as well as decrease the extent of system resources needed [34].

#### Factors preventing TWH initiatives

Lack of activities and resources dedicated to the workplace well-being, both in terms of time available for meetings, having sufficient staff to participate in the programs and funding to implement changes, together with the difficulty in creating a culture of well-being as an integral part of the company vision, inadequate workload management and flexibility in work planning, as well as the employees’ mindset outside workplace were identified as challenging issues for an effective TWH application [16, 27, 30, 32]. Specifically referring to the food sector, the complex relationships between the parent employer and the client/host company, as well as competing priorities within the specific parent employee have been recognized as possible obstacles [32]. Moreover, among factors preventing TWH initiatives there were the difficulties in the identification of safety and health priorities and a poor participatory culture. In the construction sector, the following aspects emerged as challenging for the TWH operationalization: the large proportion of small employers often without human resources and safety professionals; limited resources; productivity prioritized over health and well-being to keep jobs on track; distributed workforce across multi-employer worksites; workers moving with the work site-to-site. From a leadership perspective, potential barriers to the TWH adoption in small businesses were an offsite workforce, the difficulty in obtaining employees engagement, and generational differences (e.g., millennials being perceived as unreliable and not working as hard as older generations) [19].

#### Priority areas of intervention

Priority areas of interventions were extrapolated from exploratory studies aimed at pointing out fundamental workplace safety and health issues and possible strategies to face them. Workers have identified the redefinition of some ergonomic and organizational principles as a possible priority area of intervention to manage accident risks. This was the case of the healthcare sector where a TWH participatory and iterative process, offered nurse directors and healthcare personnel the possibility to identify and apply an inspection tool able to provide information on environmental hazards and unsafe practices to inform recommendations for implementing ergonomic solutions to reduce injury risk [12]. In the construction sector, understanding toxic effects caused by the exposure to unknown chemicals in the air and ground was pointed out as an area that needed assessment due to the possible long-term health consequences and stress that could cause in workers [22]. In this scenario, the TWH intervention would allow to remediate the prioritized problematic working condition, implementing changes to company policies, programs and practices, and then communicating these changes back to workers. Focus groups and surveys could be effective instruments for addressing physical and psychological obstacles as well as for promoting success factors to maintain a good health and safety climate [11].

Another area of intervention concerned the management of mental health issues, both related to work and private life [29]. In this view, TWH operationalization may be helpful in adequately managing the risk of suicide and post-traumatic stress in the correctional field informing series of applied research to practice meetings [24], and sustaining a healthy emotive expression to foster and promote the workers’ well-being [33]. Additionally, work-related stress was reported as an issue to be addressed in TWH interventions in different workplace contexts [11, 29], including the above-mentioned correctional reality [33], together with the promotion of sleep hygiene and fatigue management [4, 29, 33]. Workload, burnout, stress, and patients’ violence have also been reported as priorities [27]. Overall, in these contexts, the TWH approach may be effective to design worker-management participatory programs to develop integrated solutions for workplace problems addressing both work organization factors and aspects of individual behavior, consistent with the principles of the TWH model.

Finally, workers also reported the importance of promoting healthier lifestyles, including a healthier diet to reach a suitable weight control [20, 33] and an adequate management of cholesterol and blood pressure levels [33] (e.g., through meal break policies and dedicated areas), together with an increase in physical activity (also supported by a facilitated access to gyms) and smoking cessation programs [4, 13, 22, 33], that can result in an overall improvement of the workforce physical health quality. To address these issues, TWH initiatives could be aimed at promoting a reduction in alcohol, caffeine, and substance uses via communication tools such as announcements, updates, and newsletters, that were also indicated as important to support healthier lifestyles [55]. Other areas individuated for interventions by both the leadership and the workforce engaged in various working sectors were the prevention of non-transmissible chronic diseases [29, 33], also creating support groups for employees affected by such pathologies [13]. Overall, the hierarchy of hazard controls, as applied to TWH, provides a useful model for categorizing the emerged themes and prioritize next steps to face them [13].

### Applicative studies

“Applicative studies” (Table 2) regarded the TWH initiatives adopted in workplaces according to the above-mentioned hierarchy of controls model. They were primarily focused on training interventions, aimed at supporting the safety and health education, as well as at encouraging personal changes, while a lack of data on elimination, substitution, and redesign of the workplace was highlighted.

#### Target dimensions

##### Studies focused on the redesign of work environments

Only one study analyzed the effectiveness of interventions aimed to redesign the work environment [38]. In this work, overweight or obese adult workers who carried out sedentary jobs at the desk received access to a portable seated elliptical machine placed underneath their desk for 16 weeks encouraging a forward–backward pedaling movement while working at the desk. This favored an increased physical activity in employees (who experienced a significant reduction in weight, fat mass, and expected circumference), an improvement in concentration while at work, and a decrease in days missed because of physical/mental health issues.

##### Studies focused on educational and encouraging TWH interventions

Educational interventions have been focused on activities relative to the information and training on principles, strategies, and practices to protect and promote the workers’ health, safety and well-being based on the TWH approach. Recipients of such TWH plans were both the workforce, the leadership as well as the administrative staff engaged in the organization of the job activities. In some cases, pre-post-test analyses were performed to assess the effectiveness of the interventions adopted.

### Recipients: workers

#### Caregivers

The COMmunity of Practice And Safety Support (COMPASS) TWH intervention was designed to reduce the injury risk and promote the health of home care workers by addressing the unique mixture of physical and psychological hazards in home-care environments [56]. In the pilot study [37], workers (n.16) met monthly in teams for education and social support using a scripted, peer-led approach addressing health promotion or occupational safety topics. Such peer-led approach creates a collaborative network characterized by willing participation of members who share work-related knowledge, develop expertise and help each other to solve problems. The intervention produced significant improvements in individual-level of well-being as measured by increased life satisfaction and reduced negative affect. Physical health assessment, similarly, showed an improvement as demonstrated by the significant increase in the meters walked during the walking test and the blood pressure changes from a pre-hypertensive range (120–129 mmHg) to a normal range (< 120 mmHg). With respect to safety improvements, consumer-employers indicated their workers spoke with them on a near-monthly basis about safety at work and reported they have been employing housekeeping tools, new transfer and bathing tools, and correcting hazards in home, thus demonstrating the effectiveness of the TWH intervention in engaging participants in safety behaviors at work. Comparable results were obtained when the same methodology was applied in a larger sample of home care workers (n.149) [39]. Significant improvements included the use of ergonomic tools or techniques for physical work, safety communication with consumer-employers, hazard correction in homes, reduction in workdays lost because of injury. At the individual level, improvements in fruit and vegetable consumption, high-density lipoprotein cholesterol, and grip strength were determined. Consumer-employers’ reports of caregiver safety behaviors also significantly improved.

#### Sedentary workers

Within workers in transportation, urban mass transit bus operators can experience a series of obesogenic conditions, including prolonged sitting; shiftwork; variable and long work hours; time-based, psychosocial, and traffic stressors; limited and/or unpredictable breaks; and limited access to healthy food options during work (if not brought from home). In this scenario, Olson et al. [51] piloted an enhanced onboarding intervention with new bus operators designed to support both their health and early job success. Enhanced activities were integrated with traditional new bus operator training processes. Operators completed up to five study visits with researchers approximately every 3–4 months during their first year of employment. A significant difference in body weight changes after 12 months of intervention was demonstrated between participants and those that were engaged only in traditional training. Effects for physical activity, sleep, and newcomer adjustment factors were mostly positively and strongly affected by the intervention.

Office employees can be exposed to hazardous levels of sedentary work that can predispose to an increased risk for multiple chronic diseases [57], obesity [58], poorer cognitive function [59, 60] and mental distress [38, 61, 62]. In this context, Carr et al. [38] demonstrated that an integrated health promotion/health protection worksite intervention (HP/HP) including a face-to-face consultation with a single staff member trained by a certified ergonomist finalized at optimizing the employees’ ergonomic workstation, activity-promoting e-mails and access to a seated active workstation was more effective in improving light intensity physical activity as well as cardio-metabolic biomarkers (weight, total fat mass, resting heart rate, body fat percentage) and work productivity outcomes (concentration at work, days missed because of health problems) compared to a health protection-only intervention (HPO) consistent exclusively in ergonomic interventions and e-mails.

#### Construction workers

Construction workers’ injuries and poor health have been associated with the high physical demands, prolonged exposure to awkward postures, whole body vibration, long working hours, and psychosocial hazards in the work environment [63–65]. While these factors are prevalent in the construction industry, the complex, hierarchical, and fissured organization of construction work provides additional challenges for implementing traditional workplace prevention programs thus making integrated approaches more successful [66]. Therefore Peters et al. [40] described the effectiveness of a TWH based “All the Right Moves” (ARM) intervention characterized by: the Soft Tissue Injury Prevention Program (StIPP), focused on implementing ergonomics practices at the site and worker level to improve musculoskeletal health; and (2) Health Week, providing integrated health coaching opportunities for individual workers to improve ergonomic practices and health behaviors (diet, physical activity, and smoking). Researchers conducted formative research to determine what characteristics needed to be considered for the success of the intervention and its implementation and performed semi-structured interviews to capture perceived benefits of the program, feasibility, and suggestions for improvement. At one month following the program, a significant improvement in ergonomic practices and a reduction in incidences of pain and injury in the intervention sites were observed compared to the control sites. At six months, an increase in recreational physical activity and a higher consumption of fruits and vegetables could be demonstrated in the intervention group. In this study, barriers to a TWH intervention fidelity and uptake were identified in fissured multiemployer worksite, itinerant nature of workers, competing production pressures, inadequate management support, and inclement weather.

Noise induced hearing loss globally remains one of the most common self-reported occupational illnesses or injuries, particularly in the construction sector [46]. In this view, a participatory TWH approach, was applied to implement the HearWell program, aimed to preserve hearing among highway maintainers, who were involved in road construction and maintenance activities and regularly exposed to high noise levels with strategies finalized to improve hearing-related attitudes and behaviors. Such participatory approach was achieved through the collaboration between supervisors and workers in the HearWell Design Teams, a committee of representatives from Health and Safety, Operations and Finance and the research staff. The goal of the integrated plan was to educate workers (maintainers and supervisors) on the noise hazard scheme, the advantages, disadvantages, and attenuation of various types of hearing protectors, and the aim of audiometric testing, together with information on the ways to reduce noise exposure and assess noise levels. A higher level of participation was thought to support the greatest improvements in attitudes and behaviors related to hearing loss prevention which could represent a more lasting solution. Such intervention showed promising results in terms of improvement in hearing climate and employment of hearing protection devices.

#### Sale workers

Inexperience, high-risk health behaviors, and lack of knowledge about hazards in work organization and environment may all favor workplace injuries in young workers, under 25-years-old. Therefore, Aryal et al. [42] explored changes in knowledge and behavior following the TWH “Promoting U through Safety and Health (PUSH)” online, self-paced safety and health training, finalized to teach young workers employed at a city park and recreation program and employees of a multinational marketplace about safety, communication, and health. Content experts in the field of OSH and HP developed the training content. A significant increase in knowledge could be demonstrated immediately after completing the training, although it decreased in both groups in the follow-up. Marketplace participants demonstrated a greater increase in knowledge, with a significantly higher score compared to the baseline, indicating retention of knowledge three months after completing the training. The PUSH was reported useful to support participants in identifying and controlling hazards in their workplace as well as to appropriately communicate with supervisors and co-workers about their rights.

#### Correctional workers

Correctional workers are often exposed to mental health stressors at work [67, 68]. These may be linked to an increased risk for anxiety, depression, and post-traumatic disorders and favor unhealthy behaviors, e.g., smoking, alcohol use, poor eating habits, and less physical exercise, that may predispose to develop chronic health conditions, such as cardiovascular disease, high blood pressure, and metabolic syndrome [69, 70]. In this context, Kotejoshyer et al. [47] evaluated the impact of a one-year peer health mentoring program for new officers based on a TWH approach. The effectiveness of the program was demonstrated by the lower risk for burnout associated with a higher mentoring frequency. Concerning physical health parameters, although hypertension and BMI worsened in both the mentees and controls, such worsening was significantly slower in the former group compared to the latter one over time.

### Leadership

#### Supervisors

A series of TWH interventions have been focused on the supervisor leadership behaviors that may impact the company organization and ultimately affect workforce outcomes.

In city utility construction departments, supervisors completed a TWH computer-based training, the Safety and Health Improvement Program (SHIP), in family- and safety-supportive behaviors followed by 2 weeks of behavior self-monitoring [36]. At the 6-month follow-up, there were improvements in family-supportive supervisory behaviors and in blood pressure control among workers. Team effectiveness and work-life effectiveness also improved among supervisors/work groups that had low baseline levels of team cohesion and leader-member exchange (e.g., poor initial relationships between supervisors and their employees). In Anger et al. [41], supervisors completed computer-based training and self-monitoring activities on team building, work-life balance, and reinforcing targeted behaviors together with scripted safety and health education. Significant improvements in family-supportive supervisory behaviours could be demonstrated. Additional significant improvements included workers’ reported frequency of daily physical activity, family and coworker healthy diet support, team cohesion, reduced sugary snacks and drinks, sleep duration, and objectively measured systolic blood pressure.

In a different occupational context, the Army and Air National Guard in US, Hammer et al. [44] demonstrated that a TWH based supervisor support training (i.e., family and sleep health supportive supervisor behaviors) improved employee job well-being (i.e., increased job satisfaction and reduced turnover intentions), and personal well-being (i.e., reduced stress before bedtime), while reduced personal and social functional impairment.

Supervisors (including employers, parents, and educators) play an important role in protecting adolescents and young adults (< 25 years) working in agriculture with poor workplace experience [45]. In this context, a TWH based online training for supervisors focused on injury prevention, health promotion and worker well-being was effective in improving their knowledge on the risks for young workers immediately after the training, although knowledge scores decreased 3 months afterwords [45]. An increased safety communication was reported, both referring to the percentage of supervisors addressing such topics with workers, and to the frequency of discussions on such issues.

#### Top management

Leadership commitment, particularly for small enterprises, is essential to include the TWH principles in the business mission and vision, allocate resources for TWH application and being role models for TWH practices [71]. Schwatka et al. [48] evaluated the effectiveness of a TWH in-person and virtual training program for small business leaders, aimed at changing leaders’ behaviors around health, safety and well-being practices. An improvement in leaders’ self-reported TWH leadership practices was determined, although they failed to report improvements in their personal health. The same group of research [50] assessed the effectiveness of a TWH development program, targeted to leadership and senior-level leadership positions of small businesses (< 500 employees) on changing organizational and workers outcomes, in a one year follow up investigation. However, no significant difference in any outcome, such as safety and health leadership practice, safety and health climate, safety and health behaviors and well-being from baseline to follow-up was reported. When Shore et al. [43] addressed the impact of TWH policies and programs implementation, such as business TWH assessment, advising and certification, as well as leadership training programs on changes in health and safety climate in diverse small businesses, marginal measurable improvements in employee perceptions of their workplace safety and health climate were determined.

The COVID-19 pandemic created workplace challenges for employee safety and health, especially in small enterprises. Brown et al. [49] examined the impact that leadership training prior to COVID-19 in businesses engaged in several different sectors including the healthcare and social assistance, educational services, public administration, arts, entertainment and recreation, construction, real estate and rental and leasing, accommodation and food service, but also in non-profit organizations had on health and safety climate, and worker well-being. No changes in perceptions were determined before and during the pandemic period and a decline in the employee well-being scores was detected between the pre-pandemic period and subsequent COVID-19 timepoints. Therefore, the hypothesis that TWH leadership training would enable businesses to maintain their pre-pandemic perceptions of safety climate and health climate, and for their employees to retain their well-being scores was not supported by the obtained results. These findings could be due to the impact that the pandemic could have had in terms of neutralization of any leadership effect on worker well-being or to an inadequate leadership training and transfer potentially related to the fact that the intervention was not designed to address the health and safety needs of workers during a global pandemic.

## Discussion

The NIOSH developed the TWH model as a holistic approach to promote and protect worker safety, health, and well-being while ensuring enterprise outcomes [72–74]. Therefore, several studies, published in the last decade, addressed the leadership and workforce perspective in TWH operationalization. Some common, transversal issues emerged as critical for implementing successful TWH initiatives and guiding decision making at each stage of the program development and operationalization.

The leadership commitment, early on into the TWH initiative development and application, was reported as essential to make worker safety, health, and well-being a clear business priority [48]. This can provide accountability and necessary resources to implement positive working conditions, in terms of safety and well-being culture and communication, behavioral support, and familiar climate. A suitable leadership commitment was also associated to a greater rate of employees’ engagement in TWH initiatives [48]. Collaborations across the organization are also important. This means that the leadership should closely work with middle, site level managers, and give workers clear opportunities to participate in planning and implementing an intervention [27]. This kind of collaboration is useful in maintaining interest and support for specifically tailored TWH operationalization, thereby helping employees’ to feel involved and part of the process and increasing participation [72]. In this view, the role of managers in promoting the participation of the workforce in TWH plans seems relevant [17]. Interestingly, a shared transformational leadership may characterize a strategic approach to favor employees in sharing responsibility for TWH, implying the need for suitable workforce health and safety training and co-workers’ support [35].

Such a workforce participatory approach ensures to develop TWH plans successfully focused on specific priority areas of intervention. This has been confirmed by the findings emerged from the reviewed studies. In fact, these were all specifically targeted on peculiar working occupational safety and health-related conditions, such as injury, ergonomics and psychosocial risks for caregivers [37, 39], noise risks in the construction sector [46], or on specific occupational populations, such as isolated and at-risk working employees [37, 39, 40, 47, 51], subcontractors [40], and young workers in high-risk contexts, like the agricultural sector [45].

In planning solutions, specific, measurable, attainable, relevant, and time-bound objectives should be pursued [72]. This may be important to develop feasible, acceptable, and effective programs that follow the principles of health equity and that can be generalizable and disseminated into specific occupational sectors [37].

The design and implementation of participatory TWH interventions can be a challenging issue in real-world settings, especially in low-wages, fast-paced, high attrition industries. All these conditions have been identified as possible barriers to the operationalization of suitable TWH initiatives, and solutions to overcome such difficulties need to be addressed in future investigations. In this view, the role of intermediates in planning and implementing safety, health, and well-being interventions should be explored, particularly in small business contexts [30].

Training resulted a core component of the leadership TWH preparedness. In the reviewed studies, training for site managers was focused on to work with employees and support familiar relationships and health quality [44]. TWH safety and health principles, the TWH hierarchy of controls, and occupational risks have been among the topics of training interventions. Training on employees, particularly new ones during orientation periods [51], was also effective in increasing their comprehension level for all hazards and the adoption of preventive measures. Unfortunately, the fact that knowledge on occupational risks was significantly increased soon after training, but not sustained at different follow-up time points, suggested the need for defining suitable timing for refreshing and/or for effectively assessing the outcomes of the interventions, with the aim to define strategies able to eventually provide lifelong benefits [42].

Evaluation and continual improvement are fundamental to the successful interorganizational and long-term TWH implementation. Both quantitative (e.g., surveys) and qualitative (e.g., focus groups, interviews, or informal conversations with workers) data should be collected to assess the outcomes from the perspective of all the figures engaged in the TWH plans, e.g., leadership, human and welfare resources, company prevention figures and employees. Additionally, also health economics parameters should be identified and employed to ascertain the efficiency and effectiveness of TWH interventions in the production and consumption of health and healthcare [75]. This inevitably requires concerted actions of medical, sociological, ecological and political expertise that can support evidence to attract interests and funding from the engaged stakeholders. Ongoing evaluation during implementation is key to provide data for making mid-course corrections, which can influence the interventions’ ultimate success in improving outcomes.

Moreover, “one-size-fits-all” approaches cannot be considered effective in the TWH application in different contexts. Strategies to adapt evidenced-based models in new populations or in a new delivery system need to be developed [34]. In this context, models should be implemented in order to achieve improvements in terms of fidelity, feasibility, acceptability, appropriateness, costs, and sustainability [34]. Additionally, the dissemination potential of the investigated approaches, in terms of repeatability and affordability, should be assessed as a critical point of evaluation, to achieve common modes of action in comparable occupational sectors.

Some limitations emerged from our review. First, as pointed out by the results obtained through the literature search strategy and the large number of excluded articles, there are objective difficulties in defining suitable parameters around TWH when conducting a search, particularly when it aims to retrieve studies on the application of the model in different workplace settings. Additionally, it is not possible to exclude that some studies, although addressing integrated models of prevention and health promotion, failed to label their interventions as TWH initiatives, thus preventing us to include them in our review.

Only few studies are available for specific job sectors. This prevents to extrapolate suitable conclusion on the tailored TWH operationalization in peculiar settings. Almost all the retrieved studies were performed in the US: a not surprising finding, considering that the TWH model was proposed in such country and so it is tailored on the specific US legal and policy context as well as on the national organization and delivery of occupational health services. This underlines the need for researching on TWH application in other international scenarios, that may be extremely different from the US, with respect to the socio-political, economic conditions, and OSH organization. Overall, this may allow to understand the difficulties encountered in the internationalization of the TWH model, as well as the possibility that different national OSH policies may yet include some theoretical aspects of the TWH approach under their core of action. In this view, in fact, while responsibility regarding OSH is of evident importance and often legislated in many countries, activities covered under the broader topic of health and wellbeing are somewhat blurred and discretionary. This may have functioned as a limiting aspect in the worldwide spreading of TWH operationalization. Additionally, although such an approach, may be beneficial for workers, employers and the overall society, its comprehensive nature demands a high level of expertise and costs for its application that could have functioned as obstacles to its prompt application in countries far from the site of origin.

The general non-randomly enrolled, single-group, pre-test/post-test studies can characterize a bias in the interpretation of the results that should be overcome in future investigations [37]. Moreover, the generic health and safety compliance assessment (yes/no answers) may be inadequate to measure behavioral changes that need to be analyzed with more sophisticated frequency scales. TWH implementation was generally evaluated through self-reported data from a sample of workers, supervisors, or leaders. Objective data (e,g., meeting documents and communication materials) should be employed in future analyses to validate participants’ responses from the baseline assessment. The present review clearly uncovered a lack of interventions focused on eliminating or reducing the risks for the health and safety of the workforce, as well as of those centered on the redesign of the workplace, maybe due to the difficulties in applying such more expensive measures compared to the educational ones. Finally, most of the reported interventions were part of research programs carried out by the NIOSH Centers of Excellence for TWH. The engagement and responsibilities of the local company prevention figures and Occupational Medicine personnel in promoting and applying TWH strategies should be more carefully explored.

## Conclusions

In conclusion, several priorities should be considered for future research in the TWH field. Longitudinal data are necessary to understand how TWH profiles in different types of business change over time. The findings retrieved in the reviewed studies should be confirmed among larger samples using multi-level methods, taking care to evaluate both the antecedents and the consequences of the applied TWH interventions in specific occupational fields. The iterative design of the interventions should be assessed as a suitable strategy to support their easy integration and sustainability over the long-term as part of a continuous improvement model adapted to meet unique contextual working factors. Emerging occupational risks, such as the psychosocial ones derived from the application of emerging technologies, the evolving work organization as well as the experience of violence in the workplace should be carefully addressed [76, 77]. In this sense, benefits may derive from systematically incorporating empirical findings, theories, and input from stakeholders in changing the program design over time, considering the diverse set of tasks, hazards and working conditions. Moreover, future studies should consider how contextual factors, including industry, business structure, geographic location, ownership, and other factors can influence the TWH implementation. Other leadership and workforce features, including age, gender, ethnicity, and identifying workforce information (e.g., diversity, industry sector, part-time, full-time) should be considered as well. Specific attention should be given to small-medium enterprises and at fissured workplace structures, where subcontractors perform most of the work, as they may face challenging issues that need to be addressed for a suitable TWH operationalization that can also support decent work conditions in high-risk contexts. Considering the recent COVID19 pandemic, it would be informative to understand which business profiles may be associated with the best employee self-reported health outcomes during emergencies. Finally, the role of the Occupational Medicine in TWH should be stressed, as well as the possibility to include interventions during the health surveillance programs in the workplaces considering the central role of the occupational physicians in supporting the safety, health, and well-being of workers [78–81].

Research in this direction may lead guidance to overcome critical issues in TWH operationalization and enhance worker well-being by informing the design of work and work environments that are safe, health-enhancing, meaningful, and fulfilling.

## Data Availability

No datasets were generated or analysed during the current study.

## Abbreviations

ARM: All the Right Moves
CBPR: Community-Based Participatory Research
COMPASS: COMmunity of Practice And Safety Support
HMP: Peer Health Mentoring Program
PUSH: Promoting U through Safety and Health
NCC: National Corrections Collaborative
NIOSH: National Institute for Occupational Safety and Health
SHIFT: Success & Health Impacts for Transit
SHIP: Safety and Health Improvement Program
StIPP: Soft Tissue Injury Prevention
TWH: Total Worker Health
